# Effect of Harvest Time on Growth and Bioactive Compounds in *Salvia miltiorrhiza*

**DOI:** 10.3390/plants13131788

**Published:** 2024-06-28

**Authors:** Zhiheng Xing, Guihong Bi, Tongyin Li, Qianwen Zhang, Patricia R. Knight

**Affiliations:** 1 Department of Plant and Soil Sciences, Mississippi State University, Starkville, MS 39762, USA; 2 Coastal Research and Extension Center, Mississippi State University, Poplarville, MS 39470, USA

**Keywords:** danshen, havest time, tanshinone I, tanshinone IIA, cryptotanshinone, salvianolic acid B

## Abstract

Danshen (*Salvia miltiorrhiza*) is a perennial medicinal plant belonging to the Lamiaceae family. It is adapted to a wide range of soil pH with the potential to serve as an alternative crop in the United States. To enhance its cultivation and economic viability, it is crucial to develop production practices that maximize bioactive compound yields for danshen. The objective of this study was to investigate the effects of different harvest times on plant growth and subsequent yields of bioactive components of danshen. Three harvest times were selected (60, 120, or 180 days after transplanting [DAT]). In general, plants harvested at 180 DAT had higher plant growth index (PGI), shoot number, shoot weight, root number, maximum root length, maximum root diameter, and root weight compared to plants harvested at 60 or 120 DAT. However, plants harvested at 60 or 120 DAT had higher SPAD (Soil Plant Analysis Development) values. Plants harvested at 120 or 180 DAT had a higher content of tanshinone I, tanshinone IIA, cryptotanshinone, and salvianolic acid B compared to those harvested at 60 DAT. This study provides insights for optimizing the time of harvest of danshen to maximize plant growth and bioactive compound production.

## 1. Introduction

Danshen (*Salvia miltiorrhiza*), also known as red sage or Chinese sage, belongs to the Lamiaceae family, and it is one of the most ancient cultivated medicinal plants [1]. The root is the part of the plant commonly used for medicinal purposes. It is often large, fleshy, and reddish-brown and is the source of bioactive compounds that are valued for their potential health benefits [2]. Danshen is native to China and other East Asian countries. It grows well in its natural habitat on hillsides, meadows, and grassy areas next to streams and forest edges. Danshen adapts to both sunlight and partial shade, and grows best in soil that is rich and damp but drains well, especially if it is sandy [3]. Danshen is used as a raw material for making traditional Chinese medicines [4], and it has a long history of use [3]. Research investigations have demonstrated that tanshinone I, tanshinone IIA, cryptotanshinone, and salvianolic acid B are primary bioactive constituents in danshen [5,6]. These compounds are believed to have antioxidant, anti-inflammatory, and cardiovascular protective effects [3,7,8]. They have been used to support heart health, promote blood circulation, and alleviate conditions like angina, high blood pressure, and certain types of strokes [9,10].

With an increasing understanding of herbal medicine and its global use, there is a growing interest in utilizing medicinal plants for alternative and supplementary treatment to prevent and manage various diseases, including cardiovascular diseases [11]. In recent years, the use of danshen as a natural remedy has significantly increased in Europe [12]. At present, most medicinal plants, including danshen, utilized in the United States are sourced from overseas, which presents a range of challenges that impact both material quality and its use in the herbal medicine industry [13]. Quality variability, transportation costs and delays, environmental impact, and supply chain vulnerabilities are relevant concerns [14]. Having reliable access to sufficient quantities of high-quality medicinal plants is of utmost importance for industries in the United States [15]. Currently, there is limited research on the cultivation of danshen in the United States.

The bioactive compounds in herbal plants are the primary source of their medicinal properties [16], and the growth and content of bioactive compounds in plants continuously change through different stages of plant growth and development stages [17]. From the emergence of buds to the vigorous growth of flowers and roots, plants undergo significant transformations, which intricately affect their medicinal quality. Recognizing these changing features is crucial for leveraging the comprehensive benefits they provide. Danshen is a perennial plant, and, as plants grow, the bioactive compounds accumulate. In a study, three varieties of danshen were grown under identical conditions. These varieties were analyzed for different compounds in their roots at various harvest times. The results revealed that compared to 2009, two varieties had lower levels of certain compounds (cryptotanshinone, tanshinone IIA, and total tanshinone) in 2008. Conversely, the third variety had higher levels of these compounds in 2008 compared to 2009 [2]. In another study, danshen plants were harvested every two weeks and the results showed that plants harvested at the end of October had the highest root dry weight and highest tanshinone IIA content; however, plants harvested at the end of August had the highest salvianolic acid B content [18]. He et al. evaluated the dry weight of danshen and the contents of tanshinone and salvianolic acid after harvesting and found that the growth rate of danshen was fast–slow–fast. The dry weight of root peaks after the descent of frost, with tanshinone and tanshionsu levels increasing in September and October. The first peak of alcohol-soluble extract occurs from May to July, and the second peak is from October to November [2]. However, the growth characteristics of danshen in the United States still need further exploration.

The objective of this study was to investigate the effect of harvest time on growth and bioactive compound content for danshen grown in Mississippi, USA.

## 2. Results

### 2.1. Plant Growth Index, Leaf SPAD Values, Shoot Number, and Root Number

Plant growth index (PGI), SPAD (Soil Plant Analysis Development), shoot number, and root number were affected by harvest time in 2020 and 2021 (Table 1). PGI, shoot number, and root number were all greatest in plants harvested at 180 days after transplantation (DAT) and least for plants harvested at 60 DAT, regardless of year. However, SPAD measurements for plants harvested 60 or 120 DAT were greater than SPAD measurements for plants harvested 180 DAT, regardless of year. Plants harvested at 180 DAT had 61.8%, and 27.3% greater PGI compared to plants harvested at 60 DAT for 2020 and 2021, respectively. Plants harvested at 180 DAT had a similar PGI to plants harvested at 120 DAT in 2021. Shoot number increased by 125 and 133% in 2020 and 2021, respectively, when harvest intervals were increased from 60 to 180 DAT. Root number was further influenced, and root number increased by 251 and 208% in 2020 and 2021, respectively, when harvest intervals increased from 60 to 180 DAT.

### 2.2. Shoot Fresh Weight, Shoot Dry Weight, Maximum Root Length, and Maximum Root Diameter

Shoot fresh weight, shoot dry weight, maximum root length, and maximum root diameter were all greater for plants harvested at 180 DAT compared to plants harvested at 60 DAT (Table 2). Increasing the harvest interval from 60 to 180 DAT increased shoot fresh weights by 109 and 107% in 2020 and 2021, respectively, while shoot dry weights increased by 98 and 104%, respectively. Maximum root length increased by 75 and 111% when harvest interval was increased from 60 to 180 DAT in 2020 and 2021, respectively, while maximum root diameter increased by 118 and 70%, respectively. However, in 2021, the maximum root diameter values for plants harvested 60 or 120 DAT were similar.

### 2.3. Root Fresh Weight and Root Dry Weight

Both root fresh and root dry weights were greatest for plants harvested at 180 DAT and least for plants harvested at 60 DAT, regardless of year (Table 3). Increasing the harvest interval from 60 to 180 DAT increased root fresh weight by 469 and 762% in 2020 and 2021, respectively. Increasing the harvest interval from 60 to 180 DAT resulted in an even more dramatic difference in root dry weights, 2069 and 2970% in 2020 or 2021, respectively.

### 2.4. Tanshinone I, Tanshinone IIA, Cryptotanshinone, and Salvianolic Acid B

**Figure 1:**
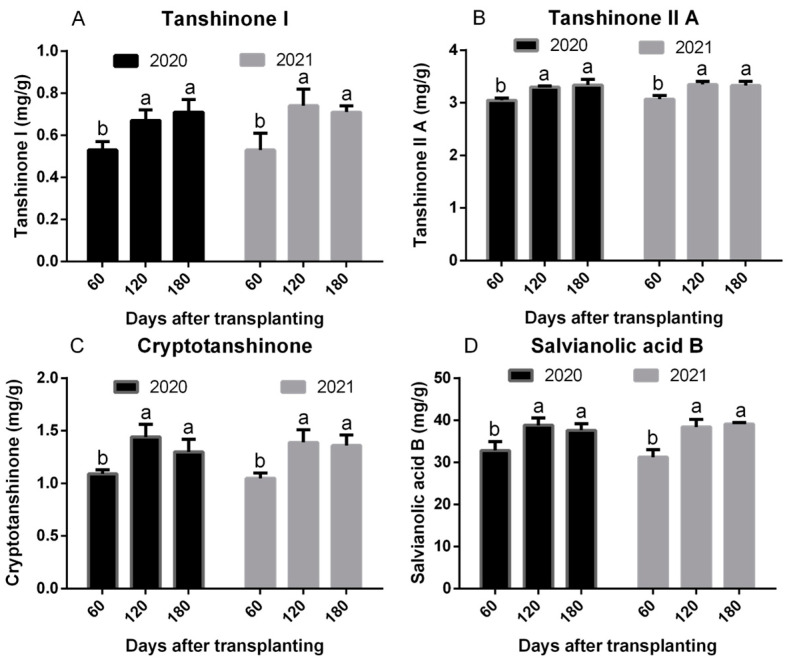
Content of tanshinone I, tanshinone IIA, cryptotanshinone, and salvianolic acid B in roots of plants growing in three harvest times in 2020 and 2021. (**A**) Tanshinone I (mg/g); (**B**) Tanshinone IIA (mg/g); (**C**) Cryptotanshinone (mg/g); (**D**) Salvianolic acid B (mg/g). Different lower-case letters suggest significant differences among three harvest times indicated by Tukey’s HSD test at *p* ≤ 0.05. Data are given as mean ± S.D.

Tanshinone I, tanshinone IIA, cryptotanshinone, and salvianolic acid B in roots were greater in plants harvested 120 or 180 DAT compared to plants harvested 60 DAT (Figure 1). Harvesting plants at 120 or 180 DAT resulted in average tanshinone I increases of 30 or 37% in 2020 or 2021, respectively, although tanshinone II A levels only increased by 9%, regardless of year. Cryptotanshinone levels averaged 26 and 31% greater when harvest intervals were increased to 120 or 180 DAT in 2020 and 2021, respectively. Levels of salvianolic acid B averaged 17 to 21% greater in plants harvested 120 or 180 DAT compared to levels in plants harvested 60 DAT in 2020 and 2021, respectively.

## 3. Discussion

The accumulation of bioactive compounds in danshen, a perennial medicinal plant, is influenced by harvest time [2], much like other medicinal plants, including ginseng [19]. In this study, harvest times of 180 DAT generally produced the largest PGI, shoot number, shoot fresh weight, and shoot dry weight. Maximizing the growth of the roots is crucial as they enable important processes such as photosynthesis, reproduction, and ecological interactions, as well as indicators of health and growth [20,21,22]. Generally, plants that grow longer before harvest can accumulate more resources and energy through photosynthesis, and absorb more nutrients and water from the soil, leading to increased leaf and stem production and larger biomass [23]. Similar results have also been reported on other plants. Subaedah et al. reported that the height of sweet corn harvested at 75 days after planting is significantly higher than that harvested 60 days after planting [24]. An increase in plant height, number of branches, and fresh weight for horehounds that were irrigated every 4 days and harvested 210 DAT compared to 80 or 150 DAT was reported by Mahmoud et al. [25]. In addition, danshen progresses through various developmental stages, including both vegetative and reproductive growth phases. Danshen flowered and seeded from September to November. PGI increased by 35.6% and 19.3% in 2020 and by 21% and 5% in 2021, between 60–120 and 120–180 DAT, respectively. Shoot numbers rose by 75% and 24% in 2020 and by 89% and 23.5% in 2021 during the same respective periods. As plants move from vegetative growth to the reproductive stage, there is rapid growth in shoot tissue [26]. In this study, the highest SPAD values were observed in plants harvested 60 or 120 DAT. Meng et al. reported SPAD values decreased as the days to harvest increased in tobacco, which supports our results [27]. Danshen harvested 60 or 120 DAT were in a vigorous growth stage with younger leaves, requiring a large amount of energy to be generated through photosynthesis, leading to an increase in chlorophyll content in the leaves [28,29]. However, leaves of plants in this study that were harvested 180 DAT were senescing and preparing for dormancy [30]. Shoot number increased as harvest time increased. Sheng reported that the number of side branches of danshen increased as the days to harvest increased [31]. Shoot biomass of turmeric increased with increasing growth time [32]. These research results are consistent with the results of this study.

The greater root number in plants harvested 180 DAT compared to 60 or 120 DAT for danshen shows the cumulative growth of roots, which is related to longer growth times, and also reported by Sheng [31]. Extended growth periods enable plants to explore the soil for water and nutrients, extending their root systems to access diverse resources, and promoting the development of a larger root system [33]. Extended growth periods provide more sunlight absorption opportunities for plants, crucial for developing both above-ground and below-ground structures. Sustained energy from photosynthesis allows for plants to allocate resources to root growth [34]. Other possible reasons for greater root biomass at longer growth times could be due to plants with longer growth periods adapting better in environments with limited or unevenly distributed resources [35]. Taller plants with increased above-ground biomass require a well-developed root system for structural support. Longer roots anchor the plant securely, preventing toppling due to the increased height [36]. Another study also observed increased root length of *Astragalus membranaceus* Bunge with increasing growth time [37]. In addition, Sheng reported that the root diameter of danshen also increased with the growth time, which is consistent with the results of this study [31]. This ongoing root growth is essential for nutrient uptake, water absorption, and overall plant health. In addition, some plants invest in root development as part of their reproductive strategies [38]. Adventitious roots or lateral roots can serve as anchor points for new shoots or offspring, contributing to the overall spread and growth of the plant population. Root propagation is also an important reproductive method of danshen [39].

The contents of tanshinone I, tanshinone IIA, cryptotanshinone, and salvianolic acid B in this study are in agreement with and are sometimes higher than those reported in other studies [2,31]. Tanshinone I content in the roots of danshen ranged from 0.02 to 0.07%, and cryptotanshinone content ranged from 0.027 to 0.27% during two years of cultivation [2,31], which is consistent with the content of this study. According to a previous study, tanshinone IIA content in the roots of danshen showed high levels during late autumn [31]. In this study, the tanshinone IIA content of Mississippi-grown danshen roots was highest at 120 or 180 DAT during the late autumn and early winter months. Considering that danshen has the highest shoot and root weight at 180 DAT, flourishing shoots, leaves, and a more extensive root system in danshen enhance photosynthesis and nutrient uptake, boosting energy and precursor availability for secondary metabolism. This improved plant health supports the synthesis and accumulation of bioactive compounds like tanshinones and phenolic acids, leading to higher compound content. The synthesis of bioactive compounds in danshen was affected by various factors. Yu et al. reported that SmAP1, SmAP2, and SmERF2 transcription factors showed increased expression levels, correlating with higher tanshinone I, tanshinone IIA, and cryptotanshinone in danshen. Malondialdehyde content and SOD, POD, and CAT activities also increased, as did key enzymes in the tanshinone biosynthesis pathway [40]. Another study found that the mevalonic acid pathway may have less influence on tanshinone accumulation than the methylerythritol 4-phosphate pathway and SmCMK could be crucial in their biosynthesis and accumulation [41]. These findings suggest that enzyme activities in bioactive compound biosynthesis fluctuate with plant age and developmental stage, peaking at specific times to optimize bioactive compound synthesis. Tanshinone and salvianolic acid are produced as part of a plant’s secondary metabolism, long growth time, or environmental stressors such as the cold in the late autumn and early winter, which allows for plants to enhance secondary metabolite synthesis, leading to higher contents of these bioactive compounds [42,43]. Golizadeh et al. reported that prolonged exposure to cold stress (6 °C) has the potential to trigger the accumulation of increased levels of bioactive compounds, such as hydrogen peroxide and malondialdehyde, in cold-tolerant wheat cultivars [44]. In this study, the temperature at 180 DAT was around 10 °C (Figures S1 and S2) for two years, which is consistent with previous research findings. At this stage, due to the decrease in temperature, plants produced more secondary metabolites. In addition to temperature, air humidity has also been reported to have a negative impact on secondary metabolism. The levels of root secondary metabolites were lower under elevated air humidity (79%) compared to control conditions (78%). This reduction included metabolites such as flavonoids, diaryl compounds, benzoic acids, monoaryl compounds, and jasmonates [45]. The relative humidity in this study was relatively low (Figures S3 and S4) in December of two years (180 DAT), which contributed to the process of secondary metabolism. Hormones have also been reported to have a certain impact on secondary metabolism. Jasmonic acid treatment releases SmJAZ8 inhibition on SmMYB97 and its downstream genes PAL1, TAT1, CPS1, and KSL1. Overexpressing SmMYB97 boosts phenolic acid and tanshinone biosynthesis [46]. Longer growth periods provide plants with more time to allocate resources to the production of bioactive compounds associated with reproductive strategies. It was reported that the flowers emitted minute quantities of the diterpene (+)-copalol, playing a pivotal role in stimulating the attraction, landing, and scent-gathering behavior of bee pollinators [47]. In our study, the content of diterpenes, which are tanshinone and salvianolic acid in danshen, reached their highest levels during the reproductive stage (120 and 180 DAT). This may be related to the role played by diterpenoid compounds.

## 4. Materials and Methods

### 4.1. Plant Materials and Cultivation

Danshen seeds were sourced from Shandong province, China (36°07′ N 120°38′ E) and stored at 4 °C until use. In March 2020 and 2021, the seeds were sown in 128-cell trays in a greenhouse at Mississippi State University (MSU, 33°29′ N 88°47′ W). One month post germination, seedlings were transplanted into 0.5 L containers and allowed to continue to grow in the greenhouse. In June of both years, plants were transplanted into larger 11.4 L containers filled with a soilless substrate (Metro-Mix^®^ 852, Sun Gro Horticulture, Agawam, MA, USA). These containers were then placed in full sun on a nursery pad at the MSU R. R. Foil Plant Science Research Center in Starkville, MS, USA.

A slow-release fertilizer, Osmocote Plus 15 N–3.9 P–10 K (15–9–12, 8–9 months; Scotts Miracle-Grow Co., Marysville, OH, USA), was applied to the plants through top-dressing at a rate of 10 g fertilizer per plant. Drip irrigation was installed to ensure the plants received appropriate water as needed. The experiment followed a randomized complete block design, with the experimental factor being harvest time. There were four replications, each comprising 15 plants (subsamples). Plants were harvested 60, 120, and 180 DAT.

### 4.2. Plant Growth

Before harvesting, measurements of plant height, Width 1, and Width 2 were taken for each plant, with five plants sampled from each harvest time. Plant height was measured from the substrate surface to the plant’s apex. Width 1 was measured at the plant’s widest point, while Width 2 represented the width perpendicular to Width 1. PGI was calculated as the average of height, Width 1, and Width 2: PGI = [height + Width 1 + Width 2]/3. Prior to each harvest, the leaf SPAD value was measured on three recently fully expanded leaves from each plant using a SPAD-502 Chlorophyll Meter (Konica Minolta, Inc., Osaka, Japan), and the SPAD value for each plant was derived from the average of three readings [30]. Subsequent to harvest, measurements of shoot number and shoot fresh weight were recorded. Shoot numbers correspond to the count of primary shoots originating from the root. Root number, maximum root length, maximum root diameter, and root fresh weight were recorded. Root number was determined by counting roots exceeding 2 mm in diameter. Maximum root length represented the longest root’s measurement [31], while maximum root diameter was assessed at the tap root’s upper middle portion [48]. Following this, shoots and roots from each plant were subjected to oven-drying at 60 °C until reaching a constant weight. The dry weights of both shoots and roots were then recorded.

### 4.3. Preparation of Danshen Extract

Dried root samples were processed by grinding them to pass through a 40-mesh (0.425 mm) sieve, utilizing a Wiley mill (Thomas Scientific, Thorofare, NJ, USA). A total of 0.5 g of the danshen powder was added into 50 mL 75% methanol aqueous solution and extracted for 30 min in an ultrasonic bath at room temperature. After the ultrasonic extraction, the resulting solution underwent filtration using a vacuum pump and a Grade 1 filter paper (GE Healthcare Bio-Sciences Corp., Marlborough, MA, USA). The filtrate was then adjusted to a fixed volume of 50 mL using a 75% methanol solution within volumetric flasks [49,50].

### 4.4. Analysis of Tanshinone I, Tanshinone IIA, Cryptotanshinone, and Salvianolic Acid B

Levels of tanshinone I, tanshinone IIA, cryptotanshinone, and salvianolic acid B within the danshen extract were determined using high-performance liquid chromatography (HPLC) equipment (1260 Infinity II series; Agilent Technologies, Wilmington, DE, USA). The danshen extract was filtered through a 0.22 μm membrane. HPLC analyses were performed using a diode array detector (G1315C Diode-array Detector, Agilent Technologies, Wilmington, DE, USA) with an injection volume of 10 μL, flow rate of 1 mL min^–1^, controlled oven temperature of 30 °C, and a C18 column [Agilent TC-C18 (2), 4.6 mm × 250 mm, 5 μm; Agilent Technologies, Wilmington, DE, USA]. Mobile phase A was 100% acetonitrile, mobile B was 0.02% phosphoric acid [51].

The program for tanshinone I, tanshinone IIA, and cryptotanshinone was 0–6 min: 61% A; 6–20 min: 61–90% A; 20–20.5 min: 90–61% A; 20.5–25 min: 61% A. Chromatograms were recorded at 270 nm. This procedure was modified slightly based on the method described in Chinese Pharmacopeia [51].

The program for salvianolic acid B was 0–20 min: 5–20% A; 20–30 min: 20–30% A; 30–40 min: 30–40% A. Chromatograms were recorded at 280 nm. This method was modified slightly based on Ren et al. [52].

The retention times for cryptotanshinone, tanshinone I, and tanshinone IIA were 12.75, 14.07, and 17.33 min, respectively, while salvianolic acid B had a retention time of 36.14 min. The contents of tanshinone I, tanshinone IIA, cryptotanshinone, and salvianolic acid B in danshen were calculated using standard curves.

### 4.5. Linear Regression and Linear Range

The regression equations, correlation coefficients, and linear ranges for the analysis of the tanshinones are shown in Table 4.

### 4.6. Reagent and Standards

Acetonitrile and phosphoric acid were obtainemind from Thermo Fisher Scientific (Waltham, MA, USA). Standards for tanshinone I, tanshinone IIA, cryptotanshinone, and salvianolic acid B were purchased from Sigma-Aldrich (St. Louis, MO, USA).

### 4.7. Statistical Analysis

The significance of the main effect was determined by analysis of variance (ANOVA) using the PROC GLM procedure. Where indicated by ANOVA, means were separated by Tukey’s Honestly Significant Difference test at *p* ≤ 0.05. All statistical analyses were performed using SAS (version 9.4, SAS Institute, Cary, NC, USA).

## 5. Conclusions

In conclusion, plants harvested at 180 DAT exhibited higher PGI, shoot number, shoot weight, root number, maximum root length, maximum root diameter, and root weight, despite having lower SPAD levels compared to those harvested at 60 and 120 DAT. Additionally, plants harvested at 120 or 180 DAT had higher concentrations of tanshinone I, tanshinone IIA, cryptotanshinone, and salvianolic acid B than those harvested at 60 DAT. Further research over two years of cultivation will aid in developing sustainable harvest time management practices to optimize danshen yield and quality.

## Figures and Tables

**Table 1:** Plant growth index (PGI), leaf SPAD value, shoot number, and root number of plants at three harvest times in 2020 and 2021.

Harvest Time (Days after Transplanting)	PGI ^x^		SPAD		Shoot Number (per Plant)		Root Number (per Plant) ^y^	
	2020	2021	2020	2021	2020	2021	2020	2021
60	27.5 ± 2.6 c ^z^	31.5 ± 1.6 b	29.8 ± 1.9 a	30.9 ± 1.3 a	3.2 ± 0.4 c	3.6 ± 0.5 c	11.8 ± 1.3 c	10.4 ± 1.1 c
120	37.3 ± 2.2 b	38.1 ± 2.8 a	31.9 ± 1.9 a	33.5 ± 2.2 a	5.6 ± 0.4 b	6.8 ± 0.8 b	19.4 ± 2.3 b	17.4 ± 1.1 b
180	44.5 ± 3.8 a	40.1 ± 3.8 a	23.9 ± 2.2 b	24.1 ± 2.5 b	7.2 ± 0.8 a	8.4 ± 1.0 a	41.4 ± 3.1 a	32.0 ± 4.5 a
*p*-value	<0.0001	<0.0001	<0.0001	<0.0001	<0.0001	<0.0001	<0.0001	<0.0001

^x^ Plant growth index (PGI) was calculated as the average of plant height, width 1 (widest points apart), and width 2 (perpendicular to width 1). [ph + width 1 + width 2]/3. ^y^ Root number was counted as the number of roots greater than 2 mm in diameter. ^z^ Different lower-case letters within a column suggest significant differences among plant densities indicated by Tukey’s HSD test at *p* ≤ 0.05. Data are given as mean ± S.D.

**Table 2:** Shoot fresh weight, dry weight, maximum root length, and maximum root diameter of plants at three harvest times in 2020 and 2021.

Harvest Time (Days after Transplanting)	Shoot Fresh Weight (g per Plant)		Shoot Dry Weight (g per Plant)		Maximum Root Length (cm) ^x^		Maximum Root Diameter (mm) ^y^	
	2020	2021	2020	2021	2020	2021	2020	2021
60	57.6 ± 5.2 c ^z^	55.5 ± 4.1 c	18.4 ± 1.6 c	18.2 ± 1.2 c	23.6 ± 3.1 c	19.2 ± 0.8 c	5.0 ± 0.4 c	6.3 ± 0.5 b
120	96.3 ± 8.4 b	93.3 ± 4.8 b	24.1 ± 1.8 b	22.7 ± 0.9 b	28.8 ± 2.8 b	34.6 ± 2.8 b	7.2 ± 0.3 b	7.3 ± 1.1 b
180	120.1 ± 6.5 a	115.0 ± 4.4 a	36.4 ± 3.1 a	37.1 ± 3.1 a	41.2 ± 2.7 a	40.6 ± 3.1 a	10.9 ± 0.9 a	10.7 ± 0.9 a
*p*-value	<0.0001	<0.0001	<0.0001	<0.0001	<0.0001	<0.0001	<0.0001	<0.0001

^x^ Maximum root length was measured as the length of the longest root. ^y^ Maximum root diameter was measured as the diameter of the thickest root. ^z^ Different lower-case letters within a column suggest significant differences among plant densities indicated by Tukey’s HSD test at *p* ≤ 0.05. Data are given as mean ± S.D.

**Table 3:** Root fresh weight and root dry weight of plants in three harvest times in 2020 and 2021.

Harvest Time (Days after Transplanting)	Root Fresh Weight (g per Plant)		Root Dry Weight (g per Plant)	
	2020	2021	2020	2021
60	46.8 ± 4 c ^z^	30.8 ± 2.2 c	3.9 ± 0.4 c	2.7 ± 0.2 c
120	146.3 ± 10.8 b	143.3 ± 10.1 b	29.8 ± 2.3 b	30 ± 6.4 b
180	266.2 ± 5.2 a	265.5 ± 11.5 a	84.6 ± 2.5 a	82.9 ± 3.5 a
*p*-value	<0.0001	<0.0001	<0.0001	<0.0001

^z^ Different lower-case letters within a column suggest significant differences among plant densities indicated by Tukey’s HSD test at *p* ≤ 0.05. Data are given as mean ± S.D.

**Table 4:** Linear regression and linear range of four compounds.

Compounds	Linear Regression	Linear Range (mg/mL)	R^2^
Cryptotanshinone	Y = 13,958X − 91.38	0.038–0.188	0.9994
Tanshinone I	Y = 3586.7X − 2.70	0.038–0.188	0.9999
Tanshinone IIA	Y = 6460.6X − 27.31	0.083–0.417	0.9998
Salvianolic Acid B	Y = 16,128X − 36.10	0.375–0.625	0.9994

X denotes concentration (mg/mL), Y denotes peak area.

## Data Availability

Data are contained within the article.

## Supplementary Materials

The following supporting information can be downloaded at: https://www.mdpi.com/article/10.3390/plants13131788/s1, Figure S1: Maximum, minimum, and average air temperatures on a daily basis in Starkville, MS, United States in 2020; Figure S2: Maximum, minimum, and average air temperatures on a daily basis in Starkville, MS, United States in 2021; Figure S3: Relative humidity, relative humidity maximum, relative humidity minimum on a daily basis in Starkville, MS, United States in 2020; Figure S4: Relative humidity on a daily basis in Starkville, MS, United States in 2021.

## References

[1] Song Z.Q., Li X.F., Wang H.G., Wang J.H. (2010). Genetic Diversity and Population Structure of *Salvia miltiorrhiza* Bunge in China Revealed by ISSR and SRAP. Genetica.

[2] He C.E., Wei J., Jin Y., Chen S. (2010). Bioactive Components of the Roots of *Salvia miltiorrhizae*: Changes Related to Harvest Time and Germplasm Line. Ind. Crops Prod..

[3] Wang B.Q. (2010). *Salvia miltiorrhiza*: Chemical and Pharmacological Review of a Medicinal Plant. J. Med. Plant Res..

[4] Fu S., Zhang J., Gao X., Xia Y., Ferrelli R., Fauci A., Guerra R., Hu L. (2010). Clinical Practice of Traditional Chinese Medicines for Chronic Heart Failure. Heart Asia.

[5] Huang J., Zhang J., Sun C., Yang R., Sheng M., Hu J., Kai J., Han B. (2023). Adjuvant Role of *Salvia miltiorrhiza* Bunge in Cancer Chemotherapy: A Review of Its Bioactive Components, Health-Promotion Effect and Mechanisms. J. Ethnopharmacol..

[6] Li Z.M., Xu S.W., Liu P.Q. (2018). *Salvia miltiorrhiza* Burge (Danshen): A Golden Herbal Medicine in Cardiovascular Therapeutics. Acta Pharmacol. Sin..

[7] Luo J., Zhang L., Zhang X., Long Y., Zou F., Yan C., Zou W. (2019). Protective Effects and Active Ingredients of *Salvia miltiorrhiza* Bunge Extracts on Airway Responsiveness, Inflammation and Remodeling in Mice with Ovalbumin-Induced Allergic Asthma. Phytomedicine.

[8] Wu Y., Xu S., Tian X.Y. (2020). The Effect of Salvianolic Acid on Vascular Protection and Possible Mechanisms. Oxid. Med. Cell. Longev..

[9] Wang L., Ma R., Liu C., Liu H., Zhu R., Guo S., Tang M., Li Y., Niu J., Fu M. (2017). *Salvia miltiorrhiza*: A Potential Red Light to the Development of Cardiovascular Diseases. Curr. Pharm. Des..

[10] Wang L.S., Yen P.T., Weng S.F., Hsu J.H., Yeh J.L. (2022). Clinical Patterns of Traditional Chinese Medicine for Ischemic Heart Disease Treatment: A Population-Based Cohort Study. Medicina.

[11] Ekor M. (2014). The Growing Use of Herbal Medicines: Issues Relating to Adverse Reactions and Challenges in Monitoring Safety. Front. Pharmacol..

[12] Xiong Y., Li M., Sun P., Liang W., Hornbeck R.G., Che X., Rao C., Zhao Y., Guo L., Huang Y. (2022). Market Access for Chinese Herbal Medicinal Products in Europe—A Ten-Year Review of Relevant Products, Policies, and Challenges. Phytomedicine.

[13] Kum K.Y., Kirchhof R., Luick R., Heinrich M. (2021). Danshen (*Salvia miltiorrhiza*) on the Global Market: What Are the Implications for Products’ Quality?. Front. Pharmacol..

[14] Golpîra H. (2020). Optimal Integration of the Facility Location Problem into the Multi-Project Multi-Supplier Multi-Resource Construction Supply Chain Network Design Under the Vendor Managed Inventory Strategy. Expert Syst. Appl..

[15] Fong H.H. (2002). Integration of Herbal Medicine into Modern Medical Practices: Issues and Prospects. Integr. Cancer Ther..

[16] Choudhary S., Zehra A., Mukarram M., Wani K.I., Naeem M., Hakeem K.R., Aftab T. (2021). Potential Uses of Bioactive Compounds of Medicinal Plants and Their Mode of Action in Several Human Diseases. Medicinal and Aromatic Plants: Healthcare and Industrial Applications.

[17] Shen B., Zhang Z., Shi Q., Du J., Xue Q., Li X. (2022). Active Compound Analysis of Ziziphus Jujuba cv. Jinsixiaozao in Different Developmental Stages Using Metabolomic and Transcriptomic Approaches. Plant Physiol. Biochem..

[18] Zhang Y. (2008). Study on the Optimal Harvesting Time of *Salva miltiorrhiza*. Res. Pract. Chin. Med..

[19] Song S.Y., Park D.H., Seo S.W., Park K.M., Bae C.S., Son H.S., Kim H.G., Lee G.H., Yoon G., Shim J.H. (2019). Effects of Harvest Time on Phytochemical Constituents and Biological Activities of *Panax Ginseng* Berry Extracts. Molecules.

[20] Das P.P., Singh K.R., Nagpure G., Mansoori A., Singh R.P., Ghazi I.A., Kumar A., Singh J. (2022). Plant-Soil-Microbes: A Tripartite Interaction for Nutrient Acquisition and Better Plant Growth for Sustainable Agricultural Practices. Environ. Res..

[21] Gupta S., Schillaci M., Walker R., Smith P.M., Watt M., Roessner U. (2021). Alleviation of Salinity Stress in Plants by Endophytic Plant-Fungal Symbiosis: Current Knowledge, Perspectives and Future Directions. Plant Soil.

[22] Lundgren M.R., Des Marais D.L. (2020). Life History Variation as a Model for Understanding Trade-Offs in Plant–Environment Interactions. Curr. Biol..

[23] Weraduwage S.M., Chen J., Anozie F.C., Morales A., Weise S.E., Sharkey T.D. (2015). The Relationship Between Leaf Area Growth and Biomass Accumulation in *Arabidopsis thaliana*. Front. Plant Sci..

[24] Subaedah S.T., Edy E., Mariana K. (2021). Growth, Yield, and Sugar Content of Different Varieties of Sweet Corn and Harvest Time. Int. J. Agron..

[25] Mahmoud A.A., Gendy A.S.H., Said-Al Ahl H.A.H., Grulova D., Astatkie T., Abdelrazik T.M. (2018). Impacts of Harvest Time and Water Stress on the Growth and Essential Oil Components of Horehound (*Marrubium vulgare*). Sci. Hortic..

[26] Poethig R.S. (2013). Vegetative Phase Change and Shoot Maturation in Plants. Curr. Top. Dev. Biol..

[27] Meng Z., Zhou Y., Yang K., Li W., Zong S., Liu F., Ma H., Zhu Y., Zhu J., Song X. (2022). Effects of Delayed Harvest on SPAD and Burnt-Sweet Mellow-Sweet Style of Flue-cured Tobacco Upper Six Leaves in Yongzhou. J. Henan Agric. Sci..

[28] Wang G., Zeng F., Song P., Sun B., Wang Q., Wang J. (2022). Effects of Reduced Chlorophyll Content on Photosystem Functions and Photosynthetic Electron Transport Rate in Rice Leaves. J. Plant Physiol..

[29] Zhang J.L., Li X.G., Xu X.H., Chen H.P., Li Y.L., Guy R.D. (2021). Leaf Morphology, Photosynthesis and Pigments Change with Age and Light Regime in Savin Juniper. Plant Biol..

[30] Donnelly A., Yu R., Rehberg C., Meyer G., Young E.B. (2020). Leaf Chlorophyll Estimates of Temperate Deciduous Shrubs During Autumn Senescence Using a Spad-502 Meter and Calibra-Tion with Extracted Chlorophyll. Ann. For. Sci..

[31] Sheng S. (2007). Cultivation and Quality Studies of Danshen (*Salvia miltiorrhiza*) in Australia. Ph.D. Thesis.

[32] Amzad Hossain M. (2010). Effects of Harvest Time on Shoot Biomass and Yield of Turmeric (*Curcuma longa* L.) in Okinawa, Japan. Plant Prod. Sci..

[33] Fageria N.K., Moreira A. (2011). The Role of Mineral Nutrition on Root Growth of Crop Plants. Adv. Agron..

[34] Schurr U., Walter A., Rascher U. (2006). Functional Dynamics of Plant Growth and Photosynthesis–From Steady-State to Dynamics–From Homogeneity to Heteroge-Neity. Plant Cell Environ..

[35] Brassard B.W., Chen H.Y., Bergeron Y. (2009). Influence of Environmental Variability on Root Dynamics in Northern Forests. Crit. Rev. Plant Sci..

[36] Osano S.N., Mwea S.K. (2015). The Effect of Strain Rate and Specimen Length on the Stress-Strain Relationship of Vegetation Roots Used in Slope Stabilization. Icastor J. Eng..

[37] Kim Y.G., Kim K.S., Chang Y.H., Yu H.S. (1996). Effects of Harvesting Time on Growth and Root Yield in *Astragalus membranaceus* Bunge. Korean J. Med. Crop Sci..

[38] Tate H.T., Page T. (2018). Cutting Propagation of *Santalum austrocaledonicum*: The Effect of Genotype, Cutting Source, Cutting Size, Propagation Me-Dium, Iba and Irradiance. New For..

[39] Sui C. (2019). Salvia miltiorrhiza Resources, Cultivation, and Breeding. The Salvia miltiorrhiza Genome.

[40] Yu W., Yu Y., Wang C., Zhang Z., Xue Z. (2021). Mechanism by which salt stress induces physiological responses and regulates tanshinone synthesis. Plant Physiol. Biochem..

[41] Yang Y., Hou S., Fan W., Lilan L., Hui N., Xia W., Wei J. (2019). Expression patterns of some genes involved in tanshinone biosynthesis in *Salvia miltiorrhiza* roots. Ind. Crops Prod..

[42] Yang L., Wen K.S., Ruan X., Zhao Y.X., Wei F., Wang Q. (2018). Response of Plant Secondary Metabolites to Environmental Factors. Molecules.

[43] Yu H., Guo W., Yang D., Hou Z., Liang Z. (2018). Transcriptional Profiles of Smwrky Family Genes and Their Putative Roles in the Biosynthesis of Tanshinone and Phenolic Acids in *Salvia miltiorrhiza*. Int. J. Mol. Sci..

[44] Golizadeh F., Kumleh H.H. (2019). Physiological Responses and Expression Changes of Fatty Acid Metabolism–Related Genes in Wheat (*Triticum aestivum*) Under Cold Stress. Plant Mol. Biol. Rep..

[45] Kharel B., Rusalepp L., Bhattarai B., Kaasik A., Kupper P., Lutter R., Mänd P., Rohula-Okunev G., Rosenvald K., Tullus A. (2023). Effects of air humidity and soil moisture on secondary metabolites in the leaves and roots of *Betula pendula* of different competitive status. Oecologia.

[46] Li L., Wang D., Zhou L., Yu X., Yan X., Zhang Q., Li B., Liu Y., Zhou W., Cao X. (2020). JA-Responsive Transcription Factor SmMYB97 Promotes Phenolic Acid and Tanshinone Accumulation in *Salvia miltiorrhiza*. J. Agric. Food Chem..

[47] Heiduk A., Haenni J.P., Meve U., Schulz S., Dötterl S. (2019). Flower Scent of *Ceropegia stenantha*: Electrophysiological Activity and Synthesis of Novel Components. J. Comp. Physiol. A Neuroethol. Sens. Neural Behav. Physiol..

[48] He C.E., Lu L.L., Jin Y., Wei J.H., Christie P. (2013). Effects of Nitrogen on Root Development and Contents of Bioactive Compounds in *Salvia miltiorrhiza* Bunge. Crop. Sci..

[49] Jiang Z., Gao W., Huang L. (2019). Tanshinones, Critical Pharmacological Components in *Salvia miltiorrhiza*. Front. Pharmacol..

[50] Yu Z.X., Zhang Y.Y., Zhao X.X., Yu L., Chen X.B., Wan H.T., He Y., Jin W.F. (2021). Simultaneous Optimization of Ultrasonic-Assisted Extraction of Danshen for Maximal Tanshinone II A and Salvianolic Acid B Yields and Antioxidant Activity: A Comparative Study of the Response Surface Methodology and Artificial Neural Network. Ind. Crops Prod..

[51] Chinese Pharmacopoeia Commission (2020). Pharmacopoeia of the People’s Republic of China 2020.

[52] Ren J., Jiang T., Li C., Gu L.H., Li J.M. (2021). Content Determination of Tanshinone and Salvianolic acid B in Zhongfeng Huichun Capsule by HPLC. Pharm. Today.

