# Supplementary material for: Effect of Harvest Time on Growth and Bioactive Compounds in Salvia miltiorrhiza
Source: Plants (Basel). 2024 Jun 28;13(13):1788. doi: 10.3390/plants13131788 (PMC11243644; doi:10.3390/plants13131788)
Supplement: Supplementary file 1 [file plants-13-01788-s001.zip › plants-2999793-supplementary.pdf]

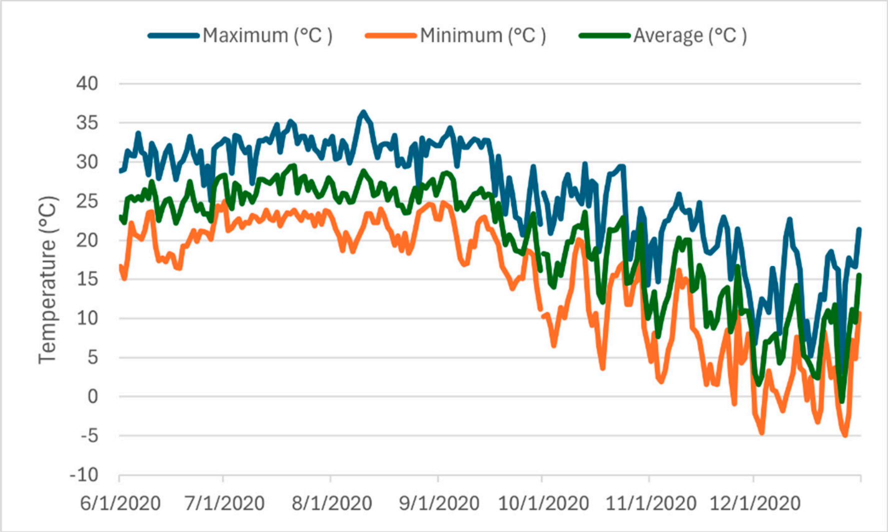

Figure S1. Maximum, minimum, and average air temperatures on a daily basis in Starkville, MS, United States in 2020. data were obtained from the website of USDA Natural Resources Conservation Service.

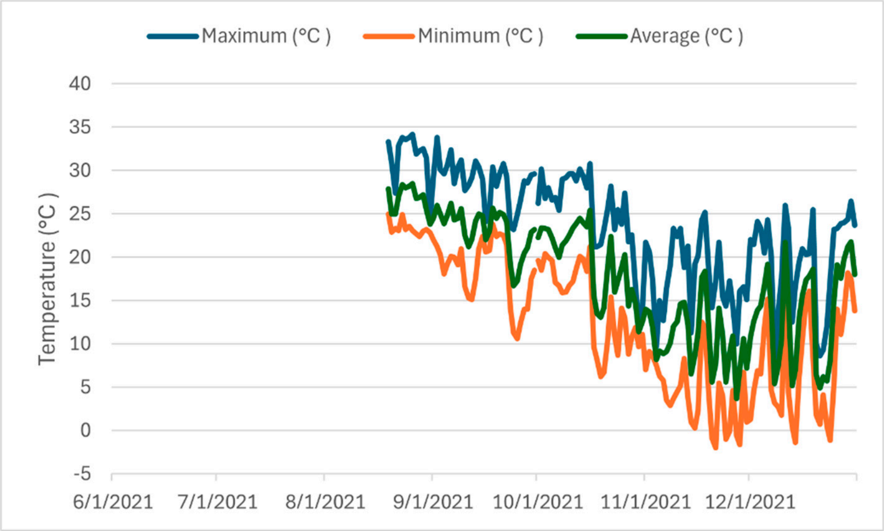

Figure S2: Maximum, minimum, and average air temperatures on a daily basis in Starkville, MS, United States in 2021. data were obtained from the website of USDA Natural Resources Conservation Service.

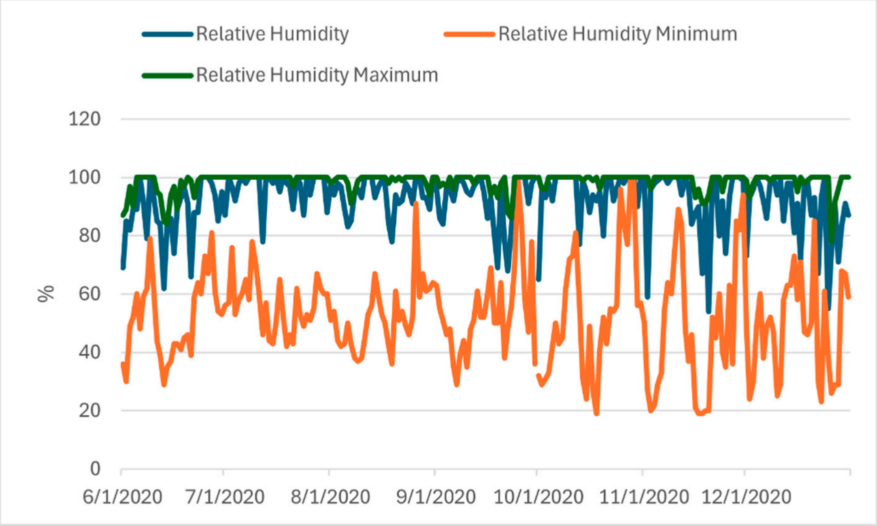

Figure S3: Relative humidity, relative humidity maximum, relative humidity minimum on a daily basis in Starkville, MS, United States in 2020. data were obtained from the website of USDA Natural Resources Conservation Service.

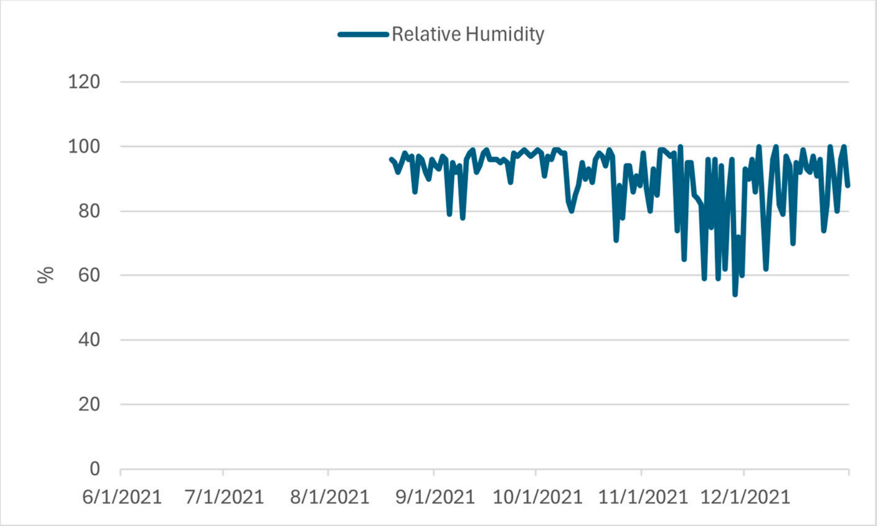

Figure S4: Relative humidity on a daily basis in Starkville, MS, United States in 2021. data were obtained from the website of USDA Natural Resources Conservation Service.
